# Effects of Chloroform on the Blood

**Published:** 1853-10

**Authors:** 


*Effects of Chloroform on the Blood.*—The April No., of the American Journal of Dental Science, for 1853, contains an article, by Mr. D. Chaumont, taken from the Monthly Journal of Medical Science, giving some experiments by this gentleman, with this article on the blood, which he thinks, go to prove, that its action, is not by asphyxia, as has been generally supposed.

Amussat, Sedilot, and others, contend, that the blood is darkened by it, while Gruby contends, that this is not only not the case, but that the venous blood is rendered by it as light as the arterial. In its direct local application, it appears to possess the property of lightening colors, reviving in some instances, the "old blood stains on linen."

M. D. Chaumont, comes to the following conclusions, he remarks.

"From the above experiments, I am inclined to think, that chloroform does not act by asphyxia, and that when this does occur, it results from the manner of its administration; that its action is directly upon the nervous centers, first upon that part connected with the perception of sensation, and lastly, on the spinal cord, or generator of motive force; that although it arterializes the color, of the venous blood out of the body. I have not been able to observe the same effect in the body.

and that, on the other hand, the arterial blood, is in no way affected in color by its inhalation."

Dr. Matthews Duncan, in connection with the same subject, remarked, that "from recent trials, he was convinced, that chloroform introduced into the stomach, could produce anæsthetic effects."

